# Diet with Diphenyl Diselenide Mitigates Quinclorac Toxicity in Silver Catfish (*Rhamdia quelen*)

**DOI:** 10.1371/journal.pone.0114233

**Published:** 2014-12-03

**Authors:** Charlene Menezes, Ignacio Ruiz-Jarabo, Juan Antonio Martos-Sitcha, Jossiele Leitemperger, Bernardo Baldisserotto, Juan Miguel Mancera, Denis Broock Rosemberg, Vania Lucia Loro

**Affiliations:** 1 Departamento de Bioquímica e Biologia Molecular, Universidade Federal de Santa Maria, Santa Maria, RS, Brasil; 2 Departamento de Biología, Facultad de Ciencias Del Mar y Ambientales, Campus de Excelencia International del Mar (CEI-MAR), Universidad de Cádiz Puerto Real, Cádiz, Spain; 3 Instituto de Ciencias Marinas de Andalucía, Consejo Superior de Investigaciones Científicas (ICMAN-CSIC), Puerto Real, Cádiz, Spain; 4 Departamento de Fisiologia e Farmacologia, Universidade Federal de Santa Maria, Santa Maria, RS, Brasil; Institute of Zoology, Chinese Academy of Sciences, China

## Abstract

In this study, the protective effects of diphenyl diselenide [(PhSe)_2_] on quinclorac- induced toxicity were investigated in silver catfish (*Rhamdia quelen*). The fish were fed for 60 days with a diet in the absence or in the presence of 3.0 mg/Kg (PhSe)_2_. Animals were further exposed to 1 mg/L quinclorac for 8 days. At the end of experimental period, fish were euthanized and biopsies from liver and gills, as well as blood samples, were collected. The cortisol and metabolic parameters were determined in plasma, and those enzyme activities related to osmoregulation were assayed in the gills. In liver, some important enzyme activities of the intermediary metabolism and oxidative stress-related parameters, such as thiobarbituric acid-reactive substance (TBARS), protein carbonyl, catalase (CAT), superoxide dismutase (SOD), glutathione S-transferase (GST), nonprotein thiols (NPSH) and ascorbic acid contents were also evaluated. Compared to the control group, quinclorac exposure significantly decreased hepatosomatic index and increased cortisol and lactate values in plasma. Moreover, the activities of fructose biphosphatase (FBPase), glucose-6-phosphate dehydrogenase (G6Pase), glycogen phosphorilase (GPase) and aspartate aminotransferase (AST) were significantly increased in liver. Quinclorac also induced lipid peroxidation while the activity of SOD, NPSH and ascorbic acid levels decreased in the liver. However, dietary (PhSe)_2_ reduced the herbicide-induced effects on the studied parameters. In conclusion, (PhSe)_2_ has beneficial properties based on its ability to attenuate toxicity induced by quinclorac by regulating energy metabolism and oxidative stress-related parameters.

## Introduction

Micronutrients are required for the normal life processes of all animals. The micronutrients, including vitamins and minerals, are important for skeletal formation, regulation of acid-base equilibrium, synthesis of hormones and enzymes, being also essential for the antioxidant defense system [1]. On the other hand, their deficiencies can cause biochemical, structural and functional changes on several metabolic parameters [2]. Selenium (Se) is an essential trace mineral for animals. However, there is a fine partition line between deficiency and toxicity. In fact, high Se concentration has been found to induce pro-oxidant effects, mainly due its ability to catalyze thiol oxidation and to generate free radicals [3], [4]. High doses of Se may also cause bioaccumulation in the trophic chain, representing a risk for organisms, but its toxicity depends essentially on its chemical structure [2].

Contrastingly, Se compounds are also able to protect fish from the toxicity of pesticides [5]–[7]. Organic forms of Se have been suggested as relevant biologic antioxidant agents in different experimental models [4], [6]. Diphenyl diselenide is an organoselenium compound and the simplest of the diaryl diselenides that has numerous pharmacological properties such as anti-inflammatory, antinociceptive, neuroprotective and antioxidant [3], [6], [8]. This compound has been used in fish diet to attempt reduce and/or attenuate the impacts of herbicides [6], [7].

Environmental exposure to herbicides may affect terrestrial and aquatic ecosystems and generate adverse effects in the human health as in aquatic organisms. The quinclorac (3,7-dichloro-8-quinolinecarboxylic acid) is a highly selective herbicide that belongs to a class of quinolone, used in turf fields and rice paddies to control some broadleaved weeds and major grass weeds [9]. This herbicide has a water solubility of 0.065 mg/L, half-life in water of around 21 days and constant of acidic ionization (pKa) of 4.34 [10]. The toxic effects of herbicides are mediated by reactive oxygen species (ROS) which can react with biological molecules and initiate oxidative damage including lipid peroxidation, protein oxidation and metabolic and enzymatic changes. Our group has already demonstrated that quinclorac exposure produces changes in the metabolic state, induces oxidative damage and reduces antioxidant status in tissues of *Leporinus obtusidens* and *Cyprinus carpio* [6], [11]. Each metabolic pathway is regulated continuously within cells in order to maintain homeostasis in the organism. Nonetheless, stressful situations can change intermediary metabolism of fish by altering key enzymes in the metabolism of carbohydrates, lipids and amino acids [12] or even osmoregulatory enzymes [13], [14]. These actions are mediated by cortisol, which provides the energy necessary to cope with the stressful situation [13].

The silver catfish, *Rhamdia quelen*, is a native freshwater fish from southern Brazil and has great economic importance. Studies have documented the physiological and biochemical responses of this species when exposed to herbicides [15], [16]. These studies showed that several biochemical parameters are altered, such as metabolic parameters, aspartate aminotransferase (AST) and alanine aminotransferase (ALT) activities, antioxidant enzymes and thiobarbituric acid-reactive substance (TBARS). Considering that herbicides may induce metabolic changes and oxidative damage in fish and that silver catfish is a species that presents a significant economic importance for human consumption, the search for potential protective molecules against herbicide-mediated toxicity and their mechanisms of action is of great importance. In this scenario, the beneficial effects of Se compounds as potential antioxidants, make these molecules candidates for the protection against quinclorac-induced toxicity. Thus, this study aimed to verify whether dietary (PhSe)_2_ protects against the detrimental effects induced by quinclorac exposure in silver catfish in terms of metabolic and oxidative-stress related parameters. To our knowledge, this is the first description of the effects of any micronutrient or herbicide over the gill H+-ATPase activity in a teleost fish and the effects of enzymes involved in the metabolism of carbohydrates, lipids and amino acids in silver catfish.

## Materials and Methods

### Chemicals

The herbicide was obtained commercially from BASF as follows: quinclorac (3,7-dichlor-oquinoline -8-carboxylic acid) (Facet; 50% purity). Malondialdehyde (MDA), 2-thiobarbituric acid (TBA), sodium dodecyl sulfate (SDS), 2,4-dinitrophenylhydrazine (DNPH), bovine serum albumin, hydrogen peroxide (H_2_O_2_) and others reagents were obtained from Sigma Chemical Co. (St. Louis, MO, USA) and commercial kits for metabolites determination from Spinreact (Barcelona, Spain). Diphenyl diselenide [(PhSe)_2_] was synthesized according to literature methods [17]. Analysis of the ^1^HNMR and ^13^CNMR spectra showed analytical and spectroscopic data in full agreement with its assigned structure.

### Fish

Juveniles specimens of silver catfish (16.32±1.87 g body mass, and 11.71±1.53 cm body length; average ± SEM) were acquired from the fish culture sector and at the Federal University of Santa Maria (UFSM) and randomly used for the experiments. Fish were randomly distributed in 250-L fiberglass boxes and acclimated to laboratory conditions prior to experimentation during 15 days. They were kept in continuously aerated tap water with a static system and with a natural photoperiod (12 h light/12 h dark). The water conditions were set as temperature: 23.0±1.0°C, pH: 7.2±0.1, dissolved oxygen: 7.0±0.5 mg/L, non-ionized ammonia: 0.3±0.01 µg/L, nitrite: 0.04±0.01 mg/L. During acclimation period the fish were fed once a day with commercial fish pellets (Supra, Brazil). Feces and pellet residues were removed by syphon and filter systems were used to maintain water quality.

### Diet preparation and Experimental design

After the acclimation period, fish were divided in two groups: control (fish fed with a diet without (PhSe)_2_, n = 24) and Se group (fish fed with a diet supplemented with 3.0 mg/Kg (PhSe)_2_, n = 24) and were fed with these diets for 60 days. The experimental diets composition was based in previous studies from our group [6]. Briefly, to obtain the diets containing (PhSe)_2_, this compound was added to control diet and all the ingredients were completely mixed by adding distilled water before further homogenization. Pellets of approximately 5 mm diameter were formed by grinding the mixture through a meat grinder. Control and (PhSe)_2_ diet pellets were stored at 4°C until they were used. In this experimental protocol fish were fed 3% biomass per day. The daily ration was divided into two equal meals fed at 09:00 and 16:00 h. The concentration of (PhSe)_2_ chosen for treating fish was based on previous studies where a dose-response curve with 1.5, 3.0 and 5.0 mg/Kg (PhSe)_2_ in the diet of silver catfish was performed. We have previously demonstrated that 3.0 mg/Kg (PhSe)_2_ did not cause overt signals of toxicity and also decreased lipid peroxidation, protein carbonylation, and increased antioxidant defenses (e.g. non-protein thiol and ascorbic acid levels). Water quality parameters were monitored daily and were maintained equal to those registered during the acclimation period.

After feeding period of 60 days, groups were sub-divided and allocated in 45-L boxes of fiberglass into four experimental groups maintained in duplicate tanks (n = 6 fish per tank, n = 12 fish per treatment): (1) control group (fish fed with a diet without (PhSe)_2_), (2) (PhSe)_2_ group (fish fed with a diet supplemented with 3.0 mg/Kg of (PhSe)_2_), (3) quinclorac group (fish fed with a diet without (PhSe)_2_ and exposed to 1 mg/L quinclorac for 8 days, (4) quinclorac + (PhSe)_2_ group (fish fed with a diet supplemented with 3.0 mg/Kg of (PhSe)_2_ and exposed to 1 mg/L of quinclorac for 8 days). Herbicide (1 mg/L) was added to the water tanks only at the beginning of the experiment, according with other experimental procedures previously described using this chemical compound [6]. Water quality did not change throughout the experimental period, and water was not replaced to avoid any disturbance, which could affects our parameters in an acute toxicity/stress response. The concentration of herbicide in water was measured at the beginning, middle and final of experimental period by high performance liquid chromatography according to Zanella et al. [18]. The limit of detection (LOD) was 0.05 µg/L and the limit of quantification (LOQ) was 0.1 µg/L. The water analysis at the end of experimental period, showed that there was a reduction of approximately 32% in the concentration of quinclorac (0.63 mg/L) in comparison to the initial value (0.93 mg/L).

The experimental procedure was authorized by the board of experimentation on Animals of the Federal University of Santa Maria (UFSM), reference number: 23081.015532/2009-31.

### Sample preparation

At the end of the exposure period, the fish were anesthetized with 50 mg/L clove oil for 3 min. After blood collection from caudal vein using heparinized syringes, the plasma obtained by centrifugation (3 min, 10 000 g, 4°C) and stored at −80°C until analysis of cortisol and metabolic parameters (glucose, lactate, triglycerides, protein and amino acids). Fish were then euthanized by spinal section and liver and gills were removed. Liver was weighed for determination of hepatosomatic index (HSI) and immediately frozen in liquid nitrogen. Branchial arches were dried in desiccant paper and also frozen in liquid nitrogen. Liver samples were used for the analysis of enzyme activities and to assess oxidative stress-related parameters and antioxidant enzymes, whereas gills biopsies were utilized for the measurement of Na^+^/K^+^-ATPase and H^+^-ATPase activities.

### Biochemical determinations in plasma samples

Cortisol levels were measured by indirect enzyme immunoassay (ELISA) adapted to microplate as described previously [19]. Glucose, triglyceride and lactate were measured using commercial kits from Spinreact (Barcelona, Spain). Total proteins were determined using bicinchoninic acid with a commercial Thermo kit using BSA (bovine serum albumin) as standard. Total α-amino acid levels were assessed colorimetrically using the nynhidrin method of Moore [20] adapted to microplates and using L-alanine as standard. All assays were performed using a Bio-Tek PowerWave 340 Microplate spectrophotometer (Bio-Tek Instruments, Winooski, VT, USA) using KCjunior Data Analysis Software for Microsoft Windows XP.

### Intermediary metabolism enzymes quantification

Frozen liver was finely minced in an ice-cold Petri dish, homogenized by ultrasonic disruption with 10 vol of ice-cold stopping-buffer containing: 50 mM imidazole (pH 7.5), 1 mM mercaptoethanol, 50 mM NaF, 4 mM EDTA, 0.5 mM PMSF and 250 mM sucrose. The homogenate was centrifuged at 10 000 g, 30 min, 4°C and the supernatant was immediately frozen using dry ice and kept at −80°C until enzyme assays. Enzyme activities were determined using a Bio-Tek PowerWave 340 Microplate spectrophotometer. Reaction rates of enzymes were determined by the increase or decrease in absorbance of NAD(P)H at 340 nm. The reactions were started by the addition of homogenates (15 µL), at a pre-established protein concentration, omitting the substrate in control wells (final volume of 275–295 µL, depending on the enzyme tested), and allowing the reactions to proceed at 37°C. The specific conditions used for hexokinase (HK), pyruvate kinase (PK), L-lactate dehydrogenase (LDH), glucose-6-phosphate dehydrogenase (G6PDH), fructose biphosphatase (FBPase), glycogen phosphorylase (GPase), alanine transaminase (ALT), aspartate transaminase (AST), glutamate dehydrogenase (GDH), and glycerol-3-phosphate dehydrogenase (G3PDH) activities were similar to those previously described [14], [21]. All enzyme assays were carried out in duplicate, at linear conditions for time and protein content detected in silver catfish (results not shown). Protein levels were assayed by triplicate as in plasma samples.

### Hepatic oxidative damage parameters

Lipid peroxidation was estimated by TBARS production, based in the reaction of malondialdehyde (MDA) with 2-thiobarbituric acid (TBA) [22]. TBARS levels were expressed as nmol MDA/mg protein. Protein carbonyl content was assessed by the method described by Yan et al. [23]. The protein carbonyl was expressed as nmol carbonyl/mg protein. All methods were previously tested and the control values are in agreement with publication of our research group [6].

### Hepatic non-antioxidants parameters

Non-protein thiols (NPSH) and ascorbic acid levels were determined according to the method of Ellman [24] and Roe [25], respectively. NPSH levels were expressed as µmol NPSH/g tissue and ascorbic acid levels as µg ascorbic acid/g tissue. These methods were also validated in our previous report [6].

### Hepatic antioxidant enzyme activities

Superoxide dismutase (SOD) activity was performed based on inhibition of the radical superoxide reaction with adrenalin as described by Misra and Fridovich [26]. The activity was expressed as U SOD/mg protein. Catalase (CAT) activity was assayed by ultraviolet spectrophotometry [27]. CAT activity was expressed in µmol/min/mg protein. Glutathione S-transferase (GST) activity was measured according to Habig et al. [28] using 1-chloro-2, 4-dinitrobenzene (CDNB) as a substrate. The activity was expressed as µmol GS-DNB/min/mg protein.

### Protein determination

Protein was determined by the Comassie blue method using bovine serum albumin as standard. Absorbance of samples was measured at 595 nm [29].

### Gill ATPase activities

Gill Na^+^/K^+^-ATPase activity was determined according to Mancera and McCormick [30]. Gill H^+^-ATPase activity was measured in the same manner as used for Na^+^/K^+^-ATPase, through from gill homogenates in the presence or absence of the specific inhibitor of the V-type H^+^-ATPase (bafilomycin A1) [31]. Na^+^/K^+^-ATPase and H^+^-ATPase activities were expressed as µmol ADP/mg protein/h.

### Statistical analysis

Descriptive statistics are presented as means ± standard error of mean (SEM). Normality and homogeneity of variances among groups were tested with the Kolmogorov-Smirnov and Levene tests, respectively. Comparisons of means was performed by one-way analysis of variance (ANOVA) followed by Duncan's multiple range tests. Results of all parameters tested form replicate tanks in each experimental condition were analyzed by Student's t-test. All data were analyzed using Statistica software (version 6.0) and the level of confidence was set at 0.05.

## Results

### Weight, length, HSI, cortisol and plasma metabolic parameters

Differences for weight and length were not detected among the treatments. A significantly lower HSI was observed in fish fed with control diet and exposed to quinclorac compared to non-exposed fish, while no changes were observed in the other groups (Table 1). Quinclorac exposure produces a 2-fold increase in cortisol levels compared to non-exposed fish, whereas (PhSe)_2_ was effective to avert the increase of this hormone when quinclorac exposure is applied. There was no difference on plasma glucose, triglyceride and amino acid between all groups tested. However, quinclorac-exposed fish showed increased lactate levels. Protein levels were lower in those specimens maintained under quinclorac + (PhSe)_2_ treatment, while the others groups did not show any variation for this parameter when compared to control group (Table 2).

**Table 1:** Growth parameters.

	Control	(PhSe)_2_	Quinclorac	Quinclorac+(PhSe)_2_
Weight (g)	24.30±2.50	31.15±2.75	24.27±1.85	32.60±5.09
Length (cm)	14.33±0.44	13.41±0.51	13.08±0.39	12.67±0.40
HSI (%)	1.13±0.07	1.17±0.05	0.87±0.02*	1.03±0.05

Weight, length, and HSI of silver catfish fed for 60 days with diets containing 0 or 3.0 mg/Kg of (PhSe)_2_ and after exposed to 1 mg/L quinclorac herbicide or to control conditions.

HSI (%)  =  liver weight/wet weight ×100. Values are means ± SEM, n = 12. (*) *P*<0.05 as compared with the control group.

**Table 2:** Metabolic parameters.

	Control	(PhSe)_2_	Quinclorac	Quinclorac+(PhSe)_2_
*Parameters*				
Cortisol	106.50±15.25	139.81±14.94	198.80±12.53*	100.28±6.87^#^
Glucose	4.15±0.55	3.74±0.42	3.97±0.36	4.98±0.45
Triglycerides	1.02±0.10	1.20±0.09	1.19±0.10	1.19±0.08
Lactate	1.12±0.28	1.40±0.43	3.26±0.53*	2.20±0.44
Protein	45.02±3.22	38.96±2.18	38.15±3.39	34.78±0.80^*^
Amino acids	12.31±1.53	11.60±1.68	9.74±1.48	13.14±1.83

Glucose, lactate, triglyceride, protein and amino acids in plasma of silver catfish fed for 60 days with diets containing 0 or 3.0 mg/Kg of (PhSe)_2_ and after exposed to 1 mg/L quinclorac herbicide or to control conditions.

Values are means ± SEM, n = 12. Cortisol is expressed as ng/mL, glucose, triglyceride, lactate and amino acids are expressed as mM and protein as mg/mL. (*) *P*<0.05 as compared with the control group. (^#^) *P*<0.05 as compared with the quinclorac group.

### Hepatic intermediary metabolism enzymes

Concerning to the carbohydrate metabolism, the measurement of HK, PK and LDH activities did not reveal significant differences when compared to control. Moreover, the FBPase and GPase activities significantly increased in the following groups: (PhSe)_2_, quinclorac, and quinclorac + (PhSe)_2_. Furthermore, the G6PDH activity enhanced in silver catfish specimens exposed to quinclorac and quinclorac + (PhSe)_2_ groups when compared to control, whereas treatment with (PhSe)_2_ *per se* did not change the activity of this enzyme (Table 3).

**Table 3:** Hepatic intermediary metabolism enzymes.

	Control	(PhSe)_2_	Quinclorac	Quinclorac+(PhSe)_2_
*Carbohydrate metabolism*				
HK	0.20±0.02	0.22±0.02	0.23±0.02	0.18±0.01
PK	4.50±0.36	3.42±0.67	4.46±0.27	4.06±0.67
LDH	0.34±0.07	0.39±0.14	0.32±0.10	0.40±0.10
FBPase	1.42±0.05	2.38±0.38*	2.16±0.24*	2.44±0.17*
G6PDH	4.80±1.68	6.40±1.80	10.35±0.88*	11.43±0.90*
GPase	1.29±0.17	2.43±0.16*	2.04±0.21*	2.19±0.29*
*Amino acid metabolism*				
ALT	2.60±0.57	4.81±1.33	2.45±0.94	2.43±0.48
AST	22.64±2.35	26.29±3.61	33.45±0.98*	32.59±3.32*
GDH	19.38±2.45	16.76±2.69	14.53±1.96	14.07±2.12
*Lipid metabolism*				
G3PDH	7.56±0.22	8.35±0.62	9.03±0.45	10.81±0.69*^#^

Enzyme activities of carbohydrate, amino acid and lipid metabolism in liver of silver catfish fed for 60 days with diets containing 0 or 3.0 mg/Kg of (PhSe)_2_ and after exposed to 1 mg/L quinclorac herbicide or to control conditions.

Values are means ± SEM, n = 12. HK, G6PDH, FBPase, PK, GPase, LDH, ALT, AST, GDH, and G3PDH are expressed as U/mg protein. (*) *P*<0.05 as compared with the control group. (^#^) *P*<0.05 as compared with the quinclorac group.

Regarding with amino acid metabolism, different treatments did not show differences on ALT and GDH activities. On the other hand, fish exposed to quinclorac (with or without (PhSe)_2_ in the diet) showed increased AST activity compared to control fish (Table 3).

Related to lipid metabolism, the G3PDH activity was significantly higher in silver catfish submitted to quinclorac + (PhSe)_2_ group and no significant difference was observed among the other groups (Table 3).

### Hepatic TBARS and protein carbonyl

**Figure 1:**
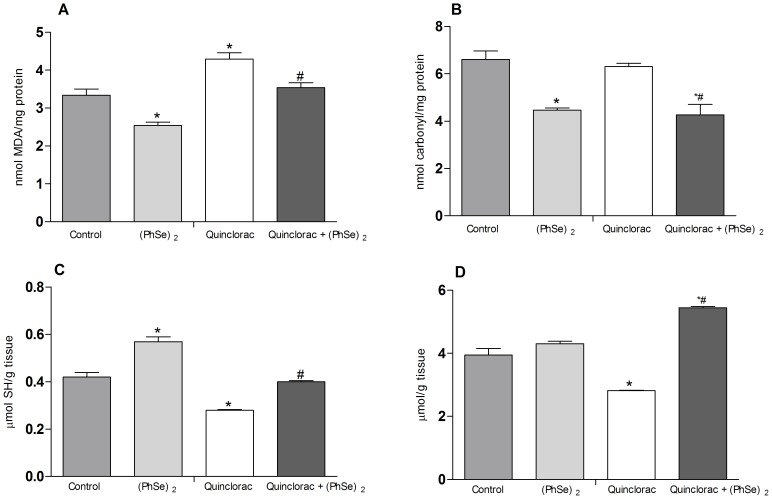
Oxidative parameters and non-enzymatic antioxidants. TBARS (A), protein carbonyl (B), NPSH (C), and ascorbic acid (D) in liver of silver catfish fed for 60 days with diets containing 0 or 3.0 mg/Kg of (PhSe)_2_ and after exposed to quinclorac herbicide or to control conditions. Data are reported as mean ± SEM (n = 12). (*) *P*<0.05 as compared with the control group. (^#^) *P*<0.05 as compared with the quinclorac group.

Fish fed with the control diet and exposed to quinclorac showed higher TBARS levels, while protein carbonyl did not significantly change (Figure 1A and 1B). Treatment with (PhSe)_2_ *per se* decreased TBARS levels and protein carbonyl of silver catfish (Figure 1A and 1B). Furthermore, (PhSe)_2_ was effective to prevent the increase of both MDA and protein carbonyl content caused by quinclorac exposure (Figure 1A and 1B).

### Hepatic NPSH and ascorbic acid levels

The NPSH and ascorbic acid levels decreased in silver catfish exposed to quinclorac when compared to control group (Figure 1C and 1D). Treatment with (PhSe)_2_ *per se* caused a significant increase in NPSH levels and did not change the ascorbic acid levels (Figure 1C and 1D). (PhSe)_2_ prevented the decrease on NPSH and ascorbic acid levels caused by quinclorac exposure (Figure 1C and 1D). Furthermore, the quinclorac + (PhSe)_2_ group showed an increased ascorbic acid levels as compared to control (Figure 1D).

### Hepatic SOD, CAT and GST activities

**Figure 2:**
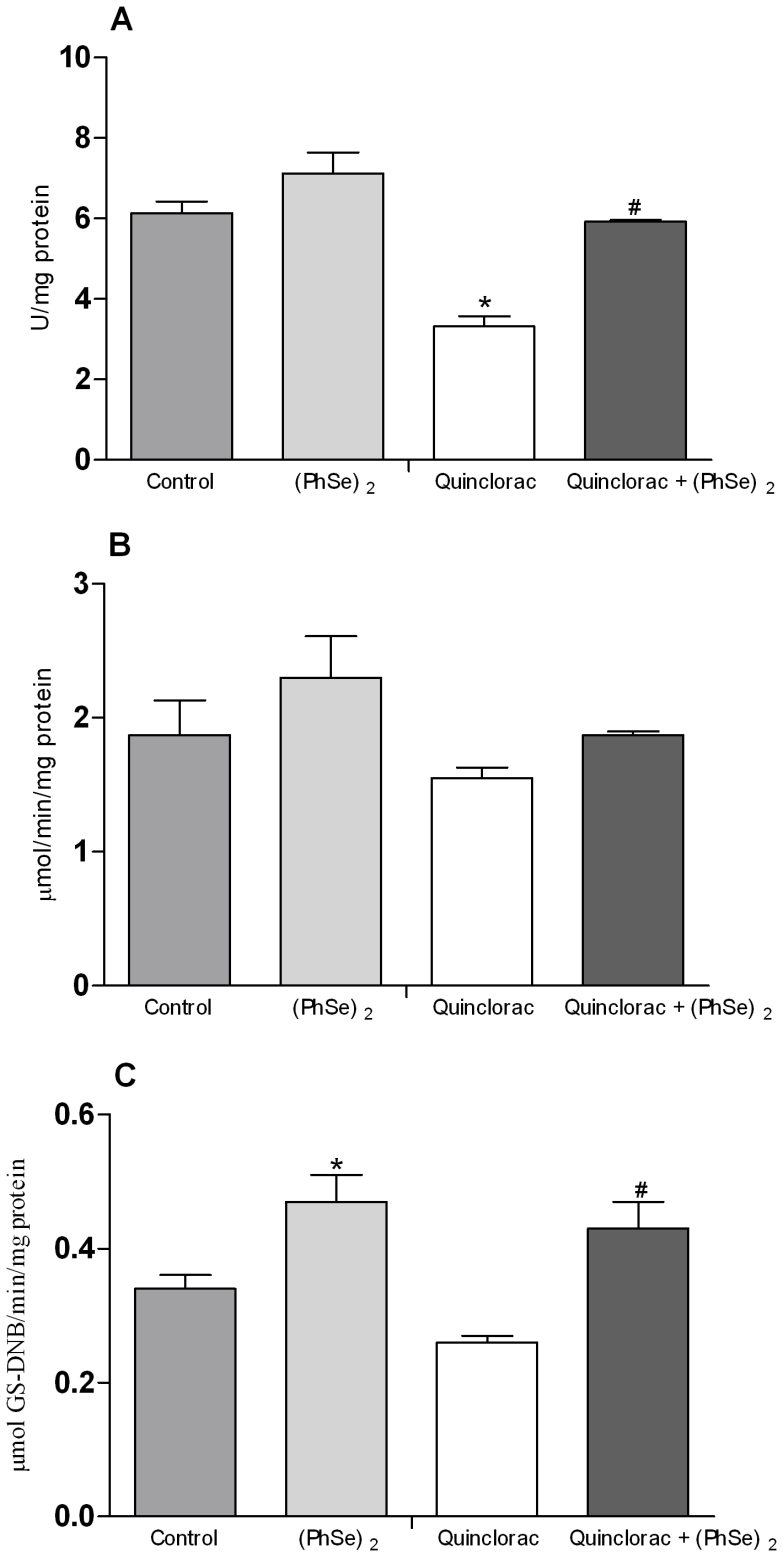
Antioxidants parameters. SOD (A), CAT (B), and GST (C) in liver of silver catfish fed for 60 days with diets containing 0 or 3.0 mg/Kg of (PhSe)_2_ and after exposed to quinclorac herbicide or to control conditions. Data are reported as mean ± SEM (n = 12). (*) *P*<0.05 as compared with the control group. (^#^) *P*<0.05 as compared with the quinclorac group.

SOD activity did not show differences in both groups of fish fed with (PhSe)_2_ respect to the control group. In addition, this activity decreased in fish exposed to quinclorac when compared to control. Moreover, (PhSe)_2_ supplementation prevented the inhibition of SOD activity caused by the herbicide (Figure 2A). CAT activity was not altered by any of the treatments tested in the present experiment (Figure 2B). Additionally, quinclorac exposure did not modify GST activity, but fish treated with (PhSe)_2_ *per se* showed an increase on GST activity. However, this enzyme activity was increased in quinclorac + (PhSe)_2_ group when compared to quinclorac group (Figure 2C).

### Gill ATPase activities

**Figure 3:**
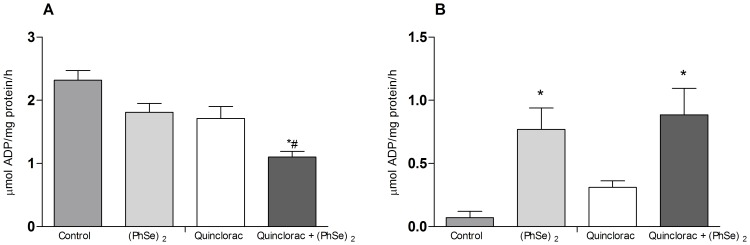
Gill ATPase activities. Na^+^/K^+^-ATPase (A) and H^+^-ATPase (B) activities in gills of silver catfish fed for 60 days with diets containing 0 or 3.0 mg/Kg of (PhSe)_2_ and after exposed to quinclorac herbicide or to control conditions. Data are reported as mean ± SEM (n = 12). (*) *P*<0.05 as compared with the control group. (^#^) *P*<0.05 as compared with the quinclorac group.

Gill Na^+^/K^+^-ATPase activity significantly decreased in the quinclorac + (PhSe)_2_ group when compared to those fish maintained as control, being also different when compared to quinclorac group (Figure 3A). Gill H^+^-ATPase activity increased in both groups fed with (PhSe)_2_, while no differences were found between quinclorac and control groups (Figure 3B). The raw data obtained from all experimental procedures are shown in Table S1.

## Discussion

In the current study, we report the protective actions of (PhSe)_2_ on the effects promoted by quinclorac, an usual herbicide used on agriculture activities, on metabolic and oxidative stress-related parameters in silver catfish. In general, quinclorac exposure caused hepatic oxidative damage, hepatotoxicity and increased both plasma cortisol and lactate levels. These data strongly suggest that quinclorac provoked stress in silver catfish and that anaerobic glycolysis occurs as a response of quinclorac effects on energy depletion. Considering that catabolism of proteins and amino acids plays a major role in total energy production in fish [32], the decrease of plasma protein levels of fish exposed to quinclorac and previously fed with (PhSe)_2_ might be due to the increased protein catabolism contributing to the organism defense against herbicide poisoning. In fact, the reduction of plasma proteins could be explained in part as a damage effect of quinclorac on hepatocyte. Importantly, (PhSe)_2_ attenuated these alterations induced by toxicant, except the increase of AST activity, which could be explained by higher metabolic mobilization produced by quinclorac compound alone as a consequence of stress pathways activation. Similarly to our results of weight and length in silver catfish, other authors also found that diets supplemented with either inorganic or organic Se forms from 1 to 2 mg/Kg levels did not affect the growth of different fish species as Atlantic salmon (*Salmo salar*) [33] or red-tailed Brycon (*Brycon cephalus*) [34]. Since HSI is a general health indicator, reflecting both the metabolic energy demand and changes in the nutritional status the decrease of HSI in fish exposed to quinclorac may result on mobilization of hepatic reserves to maintain homeostasis during stressful conditions. Additionally, this fact could also result from pesticides induced hepatic peroxisome proliferation, depleting the liver relative weight [5]. The maintenance of HSI in fish exposed to quinclorac and fed with (PhSe)_2_ suggests that Se supplementation contributes to hepatic protection.

The liver is a central organ in the metabolic processes that is responsible for various functions associated with the metabolism of contaminants. Additionally, it is also the main detoxifying organ and reacts mainly after stress caused by toxic substances [35]. Liver also has an important role in carbohydrate, protein and lipid metabolism. The activities of key metabolic enzymes can indicate the metabolic status and its ability to modify their activities against changes in environmental and dietary conditions. Due to the alterations observed in liver metabolism, fish exposed to quinclorac and quinclorac + (PhSe)_2_ showed increased hepatic gluconeogenesis favored by increased in FBPase activity, and increased power generation by high activity of the pentose phosphate shunt (G6PDH activity). In addition, the glucose produced in liver was apparently exported to other tissues, maintaining plasma glucose levels at the same time. The increased of GPase activity in all experimental groups could be associated with an increase of glycogenolysis helping to maintain glucose homeostasis. Concerning lipid metabolism, we suggest that the increase of G3PDH observed in quinclorac + (PhSe)_2_ group could be related with gluconeogenesis from glycerol for glucose production, which presumably is still needed in other tissues.

Stressful situations also induce modifications in the oxidative balance of fish. Quinclorac exposure caused hepatic oxidative damage, as demonstrated by increase in lipid peroxidation. Even so, (PhSe)_2_ normalized this parameter and it was also responsible for the reduction of protein carbonyl content exhibit a protective action in fish exposed to herbicide. These results suggest that the antioxidant activity of (PhSe)_2_ is involved in protecting against hepatic damage induced by quinclorac exposure in fish. Regarding pesticides, hepatic oxidative damage associated to an increase in lipid peroxidation was previously reported in different fish species as red-tailed Brycon [5] and silver catfish [16]. The biological systems have several mechanisms to counteract the damage caused by reactive species. Antioxidant enzymes such as SOD, CAT, GPx and non-enzymatic antioxidants such as NPSH and ascorbic acid are the most important antioxidant defenses in biological systems [36]. Importantly, we also showed that (PhSe)_2_ restored NPSH and ascorbic acid levels in liver of fish exposed to quinclorac, reinforcing the protective role of this organoselenium compound against the oxidative damage induced by herbicide. Similar results have been also described by our group, which reinforces the idea that (PhSe)_2_ could exert protective effects in fish [6].

SOD plays an important role in the dismutation of superoxide radicals to form hydrogen peroxide and molecular oxygen, while CAT is an enzyme that converts the peroxide to molecular oxygen and water. In the present study, we verified an imbalance of SOD/CAT activities induced by quinclorac exposure, which could be associated to an increase in reactive oxygen species culminating in oxidative stress. Although the mechanisms still require to be elucidated, we postulate that the antioxidant properties of (PhSe)_2_ may be involved in recovery the liver SOD activity, by increasing activity near to control values. Previous studies showed that Se supplementation increases the activity of some antioxidant enzymes in different fish species as carp or silver catfish [6], [7], [37]. GST is a detoxifying enzyme that catalyzes the conjugation of a variety of electrophilic substrates to the thiol group of GSH, producing less toxic forms [36]. (PhSe)_2_ increased the GST activity in the liver of fish exposed to quinclorac suggesting a plausible mechanism by which (PhSe)_2_ acts as a protective agent in the liver of fish. Furthermore, studies have reported that the pharmacological activity of (PhSe)_2_ mainly involves its interaction with thiol groups with a generation of intermediate selenol groups [38]. These selenol-selenolate groups can decompose H_2_O_2_ and lipid peroxides formed during the propagation phase of lipid peroxidation [3]. Our data showed that the dietary (PhSe)_2_ prevented the quinclorac-induced decrease in SOD activity and increased GST activity in silver catfish. Since quinclorac exposure did not alter GST, we suggest that the increased enzyme activity in quinclorac + (PhSe)_2_ group could be a response to selenium treatment. Previous report demonstrated that juvenile carp fed with low and high selenium commercial diets may present distinct Se levels in liver and kidney, and alterations in enzymatic antioxidant defenses, such as SOD, CAT, GST and GPx in these tissues, depending on the time of treatment [37]. Moreover, studies of our group showed that (PhSe)_2_ may play a protective role against quinclorac and clomazone-mediated toxicity in carp and silver catfish by modulating parameters oxidative stress [6], [7]. In the same context, the data presented in the current study reinforce the idea that the protective role of (PhSe)_2_ against herbicide-mediated toxicity is attributed at least in part, by regulation of fish antioxidant enzymatic defenses.

Gill H^+^-ATPase acts together with the Na^+^/K^+^-ATPase to maintain the internal homeostasis by the transport of ions across the gills epithelium [39]. The Na^+^/K^+^-ATPase (NKA) is the most important osmoregulatory enzyme in teleost fish [39]. The decreased activity of the NKA in quinclorac + (PhSe)_2_ group might be related to the decrease of cortisol release as well as a possible interaction between those two substances in gills, or could be interpreted as a diminished metabolic resources to cope with ion regulation. As discussed in the present work, the quinclorac + (PhSe)_2_ group presented an enhancement on gluconeogenic pathways in order to obtain enough energy to cope with the stressful situation, adversely affecting the energy required to maintain the NKA activity. Otherwise, the pivotal mechanisms of ion uptake in hypo-osmotic environments in the gills are the H^+^-ATPase and a Na^+^/H^+^ exchanger (NHE) among other enzymes [40]. Dietary (PhSe)_2_ increased H^+^-ATPase activity in silver catfish, but no effects could be associated to quinclorac using this methodology. The modification of this enzyme in the groups fed dietary Se could be associated to an excess of antioxidant defenses in epithelium cells of the gills, as occurs in the liver of this species when looking at the increased GST activity and NPSH levels or decreased protein carbonyl. This fact could be related to a change in the cytoplasm pH, which will change the substrates of the H^+^-ATPase enzyme.

In conclusion, our findings show the potential mechanisms by which quinclorac induces deleterious effects in fish, mainly by inducing stressful responses, altering the redox profile, and modulating enzymes of energy metabolism. Moreover, we also demonstrate that dietary (PhSe)_2_ mitigates the effects of quinclorac exposure, reducing oxidative damage and increases the activities of antioxidant enzymes and ascorbic acid levels. Therefore, diet containing (PhSe)_2_ might be beneficial in protecting fish to quinclorac intoxication. However, further studies are needed to elucidate the exact mechanisms by which (PhSe)_2_ exerted its antioxidant properties, since this organoselenium compound could be a promising alternative to minimize disorders associated with herbicide exposure in fish.

## Supporting Information

Table S1 **Raw data of the growth rate and biochemical experiments of silver catfish fed for 60 days with diets containing 0 or 3.0 mg/Kg of (PhSe)_2_ and after exposed to quinclorac herbicide or to control conditions.**
(DOCX)

## References

[1] Kim KWK, Wang X, Choi SM, Park GJ, Koo JW, et al (2003) No synergistic effects by the dietary supplementation of ascorbic acid, α-tocopheryl acetate and selenium on the growth performance and challenge test of *Edwardsiella tarda* in fingerling Nile tilapia, *Oreochromis niloticus* L. Aquac Res 34:1053–1058.

[2] Hamilton SJ (2004) Review of selenium toxicity in the aquatic food chain. Sci Total Environ 326:1–31. 15142762 10.1016/j.scitotenv.2004.01.019

[3] Nogueira CW, Rocha JBT (2010) Diphenyl diselenide a Janus-Faced molecule. J Braz Chem Soc 21:2055–2071.

[4] Nogueira CW, Rocha JBT (2011) Toxicology and pharmacology of selenium: emphasis on synthetic organoselenium compounds. Arch Toxicol 85:1313–1359. 21720966 10.1007/s00204-011-0720-3

[5] Monteiro DA, Rantin FT, Kalinin AL (2009) The effects of selenium on oxidative stress biomarkers in the freshwater characid fish matrinxã, *Brycon cephalus* (Gunther, 1869) exposed to organophosphate insecticide Folisuper 600 BR (methyl parathion). Comp Biochem Physiol C 149:40–49. 10.1016/j.cbpc.2008.06.012 18655848

[6] Menezes CC, Leitemperger J, Santi A, Lópes T, Veiverberg CA, et al (2012) The effects of diphenyl diselenide on oxidative stress biomarkers in *Cyprinus carpio* exposed to herbicide quinclorac (Facet). Ecotoxicol Environ Saf 81:91–97. 22608528 10.1016/j.ecoenv.2012.04.022

[7] Menezes C, Leitemperger J, Toni C, Santi A, Lópes T, et al (2013) Comparative study on effects of dietary with diphenyl diselenide on oxidative stress in carp (*Cyprinus carpio*) and silver catfish (*Rhamdia* sp.) exposed to herbicide clomazone. Environ Toxicol Pharmacol 36:706–714. 23892285 10.1016/j.etap.2013.07.002

[8] Barbosa NBV, Rocha JBT, Wondracek DC, Perottoni J, Zeni G, et al (2006) Diphenyl diselenide reduces temporarily hyperglycemia: possible relationship with oxidative stress. Chem Biol Interact 163:230–238. 16965767 10.1016/j.cbi.2006.08.004

[9] Sunohara Y, Shirai S, Yamazaki H, Matsumoto H (2011) Involvement of antioxidant capacity in quinclorac tolerance in *Eleusine indica* . Environ Exp Bot 74:74–81.

[10] Barceló D, Hennion MC (2002) Techniques and instrumentation analytical chemistry. In: Barceló D, Hennion MCeditors. Trace determination of pesticides and their degradation products in water. Elsevier, Amsterdam. pp.1–89.

[11] Pretto A, Loro VL, Menezes C, Moraes BS, Reimche GB, et al (2011) Commercial formulation containing quinclorac and metsulfuron-methyl herbicides inhibit acetylcholinesterase and induce biochemical alterations in tissues of *Leporinus obtusidens* . Ecotoxicol Environ Saf 74:336–341. 21036398 10.1016/j.ecoenv.2010.10.003

[12] Moon TW, Foster GD (1995) Tissue carbohydrate metabolism, gluconeogenesis and hormonal and environmental influences. In:Hochachka PW, Mommsen TPeditors.Metabolic biochemistry, biochemistry and molecular biology of fishes, vol 4. Elsevier, Amsterdam. pp.65–100.

[13] Mommsen TP, Vijayan MM, Moon TW (1999) Cortisol in teleosts: dynamics, mechanisms of action, and metabolic regulation. Rev Fish Biol Fish 9:211–268.

[14] Laiz-Carrión R, Martín del Río MP, Míguez JM, Mancera JM, Soengas JL (2003) Influence of cortisol on osmoregulation and energy metabolism in gilthead sea bream *Sparus aurata* . J Exp Zool 298A:105–118. 10.1002/jez.a.10256 12884272

[15] Glusczak L, Miron DS, Moraes BS, Simões RR, Schetinger MRC, et al (2007) Acute effects of glyphosate herbicide on metabolic and enzymatic parameters of silver catfish (*Rhamdia quelen*). Comp Biochem Physiol C 146:519–524. 10.1016/j.cbpc.2007.06.004 17716950

[16] Menezes CC, Loro VL, Fonseca MB, Cattaneo R, Pretto A, et al (2011) Oxidative parameters of *Rhamdia quelen* in response to commercial herbicide containing clomazone and recovery pattern. Pestic Biochem Physiol 100:145–150.

[17] Paulmier C (1986) Selenium Reagents and Intermediates in Organic Synthesis. Pergamon. Books, Oxford, UK, pp.84–116.

[18] Zanella R, Primel EG, Gonçalves FF, Kurz MHS, Clóvia CM (2003) Development and validation of a high-performance liquid chromatographic procedure for the determination of herbicide residues in surface and agriculture waters. J Separ Sci 26:935–938.

[19] Martos-Sitcha JA, Wunderink YS, Straatjes J, Skrzynska AK, Mancera JM, et al (2014) Different stressors induce differential responses of the CRH-stress system in the gilthead sea bream (*Sparus aurata*). Comp Biochem Physiol A Mol Integr Physiol 177:49–61. 25088183 10.1016/j.cbpa.2014.07.021

[20] Moore S (1968) Amino acids analysis: Aqueous dimethyl sulfoxide as solvent for the ninhydrin reaction. J Biol Chem 243:6281–6283. 5723468

[21] Polakof S, Arjona FJ, Sangiao-Alvarellos S, Martín del Río MP, Mancera JM, et al (2006) Food deprivation alters osmoregulatory and metabolic responses to salinity acclimation in gilthead sea bream *Sparus auratus* . J Comp Physiol B 176:441–452. 16432730 10.1007/s00360-006-0065-z

[22] Buege JA, Aust SD (1978) Microsomal lipid peroxidation. Meth Enzymol 52:302–309. 672633 10.1016/s0076-6879(78)52032-6

[23] Yan LJ, Traber MG, Packer L (1995) Spectrophotometric method for determination of carbonyls in oxidatively modified apolipoprotein B of human low-density lipoproteins. Anal Biochem 228:349–351. 8572318 10.1006/abio.1995.1362

[24] Ellman GL (1959) Tissue sulfhydryl groups. Arch Biochem 82:70–77. 13650640 10.1016/0003-9861(59)90090-6

[25] Roe JH (1954) Methods of biochemical analysis. In:Glick Deditor. Interscience Publishers. New York, pp.115–139.

[26] Misra HP, Fridovich I (1972) The role of superoxide anion in the auto-oxidation of epinephrine and a simple assay for superoxide dismutase. J Biol Chem 247:3170–3175. 4623845

[27] Nelson DP, Kiesow LA (1972) Enthalphy of decomposition of hydrogen peroxide by catalase at 25°C (with molar extinction coefficients of H_2_O_2_ solution in the UV). Anal Biochem 49:474–478. 5082943 10.1016/0003-2697(72)90451-4

[28] Habig WH, Pabst MJ, Jacoby WB (1974) Glutathione S-transferase, the first enzymatic step in mercapturic acid formation. J Biol Chem 249:7130–7139. 4436300

[29] Bradford MMA (1976) A rapid and sensitive method for the quantification of microgram quantities of protein utilizing the principle of protein-dye binding. Anal Biochem 72:248–254. 942051 10.1016/0003-2697(76)90527-3

[30] Mancera JM, McCormick SD (2000) Rapid activation of gill Na^+^-K^+^-ATPase in the euryhaline teleost *Fundulus heteroclitus* . J Exp Zool 287:263–274. 10951386

[31] Bowman EJ, Siebers A, Altendof K (1988) Bafilomycins - a class of inhibitors of membrane ATPases from microorganisms, animal-cells, and plant-cells. Proceedings of the National Academy of Sciences of the United States of America 85:7972–7976. 2973058 10.1073/pnas.85.21.7972 PMC282335

[32] David M, Mushigeri SB, Shivakumar R, Philip GH (2004) Response of *Cyprinus carpio* (Linn.) to sublethal concentration of cypermethrin: alterations in protein metabolic profiles. Chemosphere 56:347–352. 15183996 10.1016/j.chemosphere.2004.02.024

[33] Lorentzen M, Maage A, Julshamn K (1994) Effects of dietary selenite or selenomethionine on tissue selenium levels of Atlantic salmon (*Salmo solar*). Aquaculture 121:359–367.

[34] Monteiro DA, Rantin FT, Kalinin AL (2007) Use of selenium in matrinxã feed, *Brycon cephalus* . Braz J Anim Health Prod 8:32–47.

[35] Salbego J, Pretto A, Gioda C, Menezes C, Lazzari R, et al (2010) Herbicide formulation with glyphosate affects growth, acetylcholinesterase activity, and metabolic and hematological parameters in piava (*Leporinus obtusidens*). Arch Environ Contam Toxicol 58:740–745. 20112104 10.1007/s00244-009-9464-y

[36] Modesto KA, Martinez CBR (2010) Roundup causes oxidative stress in liver and inhibits acetylcholinesterase in muscle and brain of the fish *Prochilodus lineatus* . Chemosphere 78:294–299. 19910015 10.1016/j.chemosphere.2009.10.047

[37] Elia AC, Prearo M, Pacini N, Dorr AJM, Abete MC (2011) Effects of selenium diets on growth, accumulation and antioxidant response in juvenile carp. Ecotoxicol Environ Saf 74:166–173. 20554323 10.1016/j.ecoenv.2010.04.006

[38] Luchese C, Nogueira CW (2010) Diphenyl diselenide in its selenol form has dehydroascorbate reductase and glutathione S-transferase-like activity dependent on the glutathione content. J Pharm Pharmacol 62:1146–1151. 20796193 10.1111/j.2042-7158.2010.01147.x

[39] McCormick SD (2001) Endocrine control of osmoregulation in teleost fish. Am Zool 41:781–794.

[40] Evans DH, Piermarini PM, Choe KP (2005) The multifunctional fish gill: dominant site of gas exchange, osmoregulation, acid-base regulation, and excretion of nitrogenous waste. Physiol Rev 85:97–177. 15618479 10.1152/physrev.00050.2003

