# Supplementary material for: Diet with Diphenyl Diselenide Mitigates Quinclorac Toxicity in Silver Catfish (Rhamdia quelen)
Source: PLoS One. 2014 Dec 3;9(12):e114233. doi: 10.1371/journal.pone.0114233 (PMC4254993; doi:10.1371/journal.pone.0114233)
Supplement: Table S1 — Raw data of the growth rate and biochemical experiments of silver catfish fed for 60 days with diets containing 0 or 3.0 mg/Kg of (PhSe)2 and after exposed to quinclorac herbicide or to control conditions. (DOCX) [file pone.0114233.s001.docx]

**Table S1:** Raw data of the growth rate and biochemical experiments of silver catfish fed for 60 days with diets containing 0 or 3.0 mg/Kg of (PhSe)_2_ and after exposed to quinclorac herbicide or to control conditions.
